# Seroprevalence of an antibody against diphtheria, tetanus, and pertussis among the elderly in Khon Kaen, Thailand

**DOI:** 10.1186/s41043-019-0186-0

**Published:** 2019-10-18

**Authors:** Teeraporn Chinchai, Nawarat Posuwan, Viboonsak Vuthitanachot, Nasamon Wanlapakorn, Yong Poovorawan

**Affiliations:** 1 Department of Microbiology, Faculty of Medicine, Srinakharinwirot University, Bangkok, 10110 Thailand; 2 Center of Excellence in Clinical Virology, Department of Pediatrics, Faculty of Medicine, Chulalongkorn University, Bangkok, 10330 Thailand; 3 Chumpare Hospital, Chum Phae, Khon Kaen, 40130 Thailand

**Keywords:** Seroprevalence, Diphtheria, Tetanus, Pertussis, Elderly

## Abstract

**Background:**

Owing to a declining birth rate and longer lifespan, the number of elderly people (≥ 60 years) in Thailand has grown rapidly. However, the elderly are at significant risk of infectious diseases because they have never been immunized, because they have not been completely immunized, or because their immunity has waned. Immunity against infectious diseases in the elderly is an important means of controlling diseases in the community. Our objective was to evaluate the seroprotective rate against diphtheria, tetanus, and pertussis in the elderly Thai population.

**Methods:**

In total, 430 healthy individuals from the northeastern region of Thailand were enrolled in this study and stratified into five age groups: 60–65, 66–70, 71–75, 76–80, and > 80 years. Serum samples were collected and quantitatively analyzed for diphtheria, tetanus, and pertussis IgG antibody by using commercial ELISA kits. For anti-diphtheria toxoid and anti-tetanus toxoid ELISA, values < 0.01 IU/ml were interpreted as seronegative, and for anti-*Bordetella pertussis* toxin ELISA, values < 5 IU/ml were interpreted as seronegative; these definitions were in accord with previous studies.

**Results:**

For diphtheria toxoid Ab, the majority of the population had antibody levels > 0.01 IU/ml. For tetanus anti-toxoid Ab, the majority of the population had antibody levels of > 0.01 IU/ml, of which approximately 34% had durable antibody protection levels (DAPL) of ≥ 1 IU/ml. Meanwhile, nearly 45% of the population had an Ab level against pertussis lower than the protectivity level.

**Conclusions:**

In total, 97.2%, 83.5%, and 55.8% of the population had a higher antibody level than the minimal protective level for diphtheria, tetanus, and pertussis, respectively. In order to prevent an outbreak of these diseases in the future, the elderly should be administered with Tdap revaccination to provide diphtheria herd immunity in the population; this will increase cocoon phenomenon for pertussis and protect the population from tetanus-prone injury.

## Background

Over the past several decades, Thailand has become one of the most successful countries in reducing its fertility level over a relatively short period of time. The total fertility rate has declined from over six births per woman in the mid-1960s to below two births per woman since the mid-1990s. During the same period, life expectancy at birth has increased from 55.2 years to 69.9 years for men and from 61.8 years to 74.9 years for women. The number of elderly people (defined as ≥ 60 years) in Thailand has grown rapidly and will continue to do so in future decades. Since 1960, the number of older people in the Thai population has increased sevenfold from approximately 1.7 million (4.9%) in 1970 to 11.2 million (17.1%) by 2017 and is expected to rise to 15.6 million (or 23.4% of the total population) by 2026. Future population aging will occur even more rapidly, with the number of older persons projected to increase to over 19.7 million by 2036, at which point they will constitute over 30% of the population. Moreover, within the next few years, the proportion of the population who are 60 year of age, and older, will outnumber the number of children aged under age 15 for the first time in Thai history [1, 2]. The elderly are at significant risk of infectious diseases because they have never been immunized, have not been completely immunized, or their immunity has waned. Despite this, some have long-lasting immunity due to natural infection. Therefore, establishing immunity against all infectious diseases in this particular population is very important in the control of disease outbreaks in the future.

Diphtheria, tetanus, and pertussis are three serious infectious diseases with an often fatal outcome. Diphtheria is an infectious disease caused by *Corynebacterium diphtheriae*, a gram-positive, uncapsulated bacillus, most often transmitted via the aerosol route. Human asymptomatic carriers are a major source of infection [3, 4]. Tetanus is a rare disease, but has a high mortality rate. Tetanus occurs by the penetration of *Clostridium tetani* spores through contaminated wounds, lacerations, and abrasions. Deep wounds, with lacerated and bruised margins, devitalized tissue, and soil contaminations are at high risk of tetanus [3, 5]. Pertussis, also known as a whooping cough, is an acute respiratory tract infection that presents as a chronic cough in most patients and has increased in incidence over recent years. Most cases of pertussis are caused by *Bordetella pertussis* [6–11]. In 1977, Thailand implemented a routine infant immunization program with two doses of the diphtheria-tetanus toxoid and whole-cell pertussis (DTP) vaccine for all infants [12]. This recommendation was changed to three doses of DTP in 1982 and four doses (at 2, 4, 6, and 18 months) in 1987. Since 1992, the national vaccine policy in Thailand has used five doses of DTP vaccine for children at the ages of 2, 4, 6, 18, and 48 months [13]. However, outbreaks of these diseases have been reported from all over the world [6, 14–17]. According to an aging society in Thailand in the near future, the present study aimed to evaluate the seroprotective rate against these three diseases in the elderly Thai population for use as criteria for the administration of vaccine boosters in the future.

## Methods

### Population study and specimen collection

**Fig. 1:**
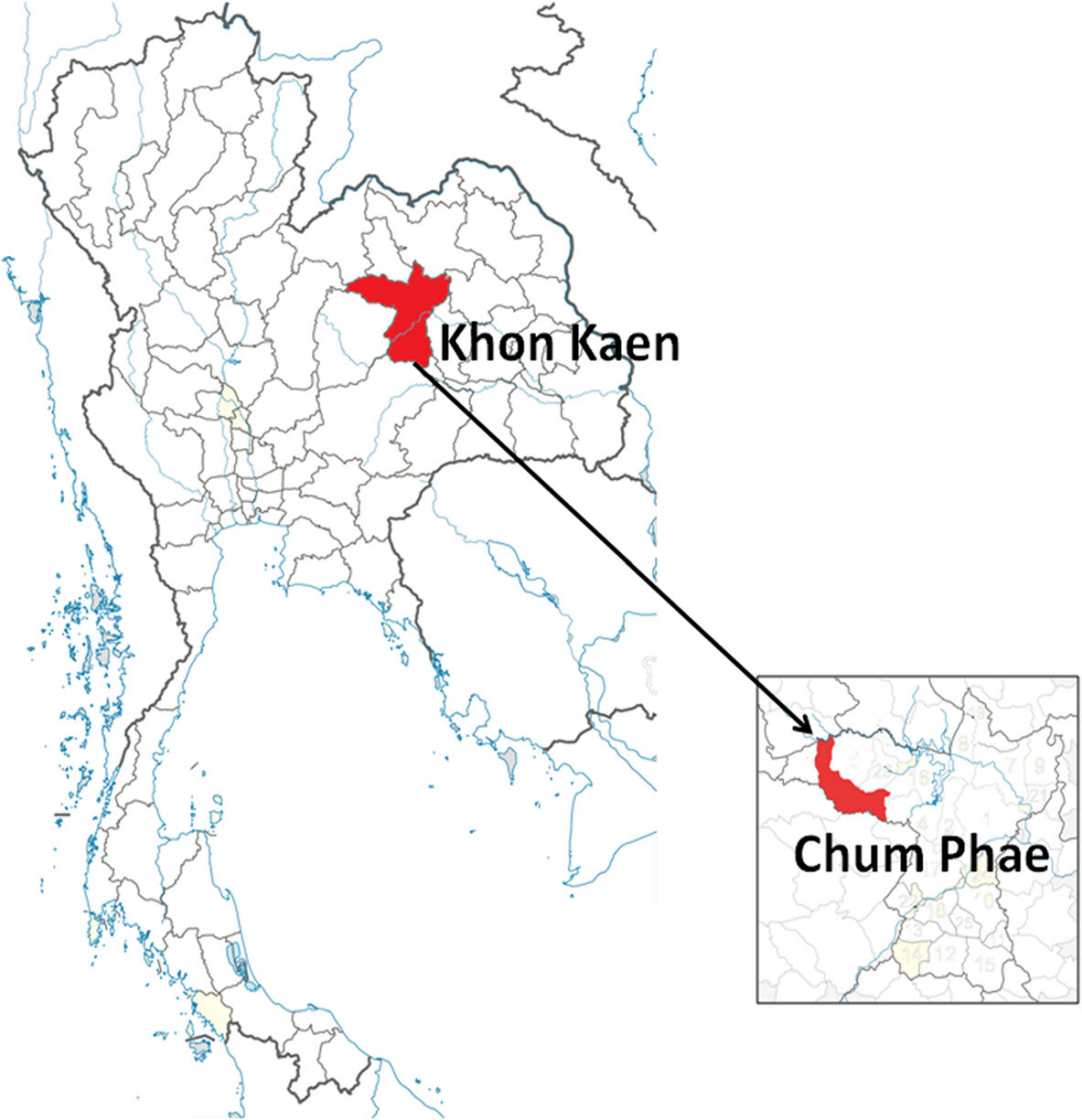
Map of Thailand showing the area from which serum samples were taken

The population under study consisted of 430 healthy individuals (123 males and 307 females), from one district of the northeastern region of Thailand (Chum Phae, Khon Kaen Province) as shown in Fig. 1. Nurses and phlebotomists visited participants at home to obtain informed consent and collect blood samples. The study protocol was approved by the Institutional Review Board of the Faculty of Medicine, Chulalongkorn University (IRB No.006/60), and the study was conducted in compliance with the principles of the Declaration of Helsinki under good clinical practice. Informed written consent was obtained from each participant. Patients were then stratified into five age groups as shown in Table 1 (aged 60 to 65, 66 to 70, 71 to 75, 76 to 80, and older than 80 years). Those who carried bedridden, acute or chronic infections, autoimmune diseases, malignancies, or immunological and hematological disorders and those who had received blood/blood components including immunoglobulin were excluded to prevent falsified results due to the distortion of immunity. Serum samples were analyzed at the Center of Excellence in Clinical Virology, Department of Pediatrics, Faculty of Medicine, Chulalongkorn University. All samples were treated anonymously.

**Table 1:** The study population enrolled in this study

Age group (years)	Total	Male	Female	*p* value^†^
61–65	104	25	79	0.18
66–70	99	23	76	
71–75	92	27	65	
76–80	70	27	43	
81+	65	21	44	

^†^Using the chi-square test

### Seroprevalence assay

Serum samples were quantitatively analyzed for diphtheria, tetanus, and pertussis IgG antibody by using commercial ELISA kits (EUROIMMUN, Lübeck, Germany) according to the manufacturer’s instructions and quantified in international units per milliliter (IU/ml). For anti-diphtheria toxoid and anti-tetanus toxoid ELISA, values < 0.01 IU/ml were interpreted as seronegative (minimum protective level is 0.01 IU/ml) [18], and for anti-*Bordetella pertussis* toxin ELISA, values < 5 IU/ml were interpreted as seronegative. These definitions were in accordance with published studies [13, 19, 20].

### Statistical analysis

Data are presented in graphs and tables showing the current seroprevalence of IgG antibody to diphtheria toxoid, tetanus toxoid, and pertussis toxin in both numbers and percentages. The geometric mean titer (GMT) was calculated from anti-diphtheria toxoid and also from anti-tetanus toxoid titer ≥ 0.01 IU/ml and anti-pertussis toxin titer ≥ 5 IU/ml by multiplying the antibody levels of individuals and taking the *n*th root of the product (where *n* was the number of observations). The analysis of antibody titers was also done using the logarithmic transformed data. The one way-ANOVA analysis was used to evaluate the level of antibodies against pertussis, diphtheria, and tetanus between the aged groups, and independent *t* test was used to compare the level of antibodies against pertussis, diphtheria, and tetanus between females and males. The chi-square test was used to evaluate the association of gender with aged groups. The *p* value less than 0.05 were considered as statistically significant. All statistical analyses were completed using STATA version 13.0.

## Results

### Study population

The study population is shown in Table 1. The number of females was greater than the number of males, in all age groups but was not significantly different.

### Seroprevalence study

IgG antibody titer was first determined in all samples; the results are described below.

### Anti-diphtheria toxoid antibody

**Fig. 2:**
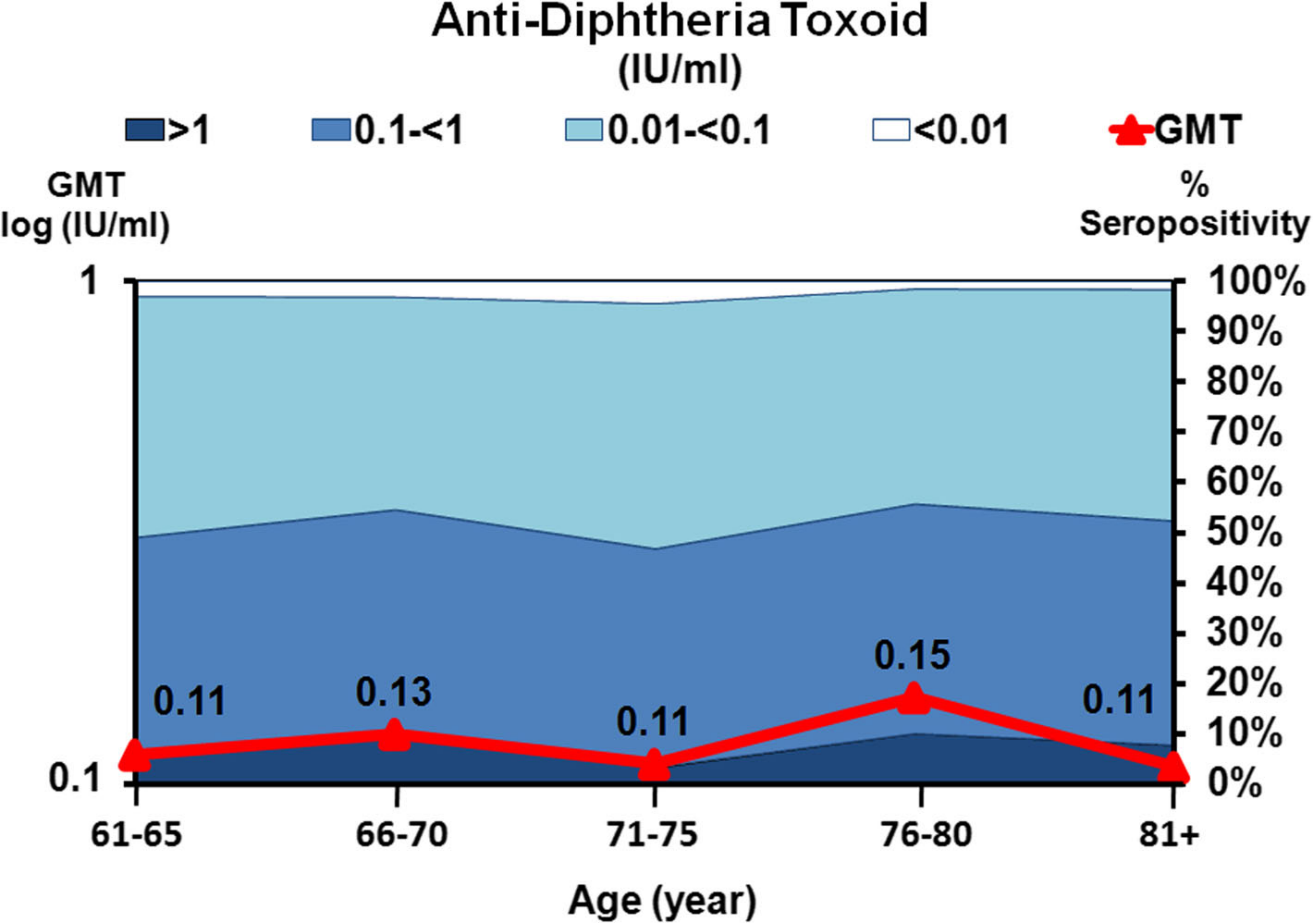
The prevalence of anti-diphtheria toxoid antibody and geometric mean titers (GMTs) in the study population. The *x*-axis represents the five age groups and the sample size in each age group. The scale on the right represents the proportion (%) of the population with positive anti-diphtheria toxoid antibody. The scale on the left represents the GMTs in each age group, with the means indicated as a red line. Antibody measurements were negative (white), 0.01 to < 0.1 IU/ml (light blue), 0.1 to < 1 IU/ml (blue), and > 1 IU/ml (dark blue)

Anti-diphtheria toxoid antibody levels in all age groups are shown in Fig. 2 and Table 2; GMT is shown in Table 3. The data were classified according to the levels of antitoxoid: < 0.01 IU/ml (susceptible), 0.01 to 0.09 IU/ml (low immunity), 0.1 to 0.99 IU/ml (satisfactory, protective level) and ≥ 1 IU/ml (long-lasting immune protection), as indicated in a previous study [20].

**Table 2:** The proportion (%) of seropositive rates across different age groups

Age (year)	Total	Seropositivity								
		Diphtheria			Tetanus			Pertussis		
		*N*	%	95% (CI)	*N*	%	95% (CI)	*N*	%	95% (CI)
61–65	104	101	97.1	93.8–100	90	86.5	79.8–93.2	53	51.0	41.2–60.8
66–70	99	96	97.0	93.5–100	83	83.8	76.4–91.2	57	57.6	47.6–67.5
71–75	92	88	95.7	91.4–99.9	72	78.3	69.7–86.9	48	52.2	41.8–62.6
76–80	70	69	98.6	95.8–100	63	90.0	82.8–97.2	44	62.9	51.3–74.4
81+	65	64	98.5	95.4–100	51	78.5	68.3–88.7	38	58.5	46.2–70.7
Total	430	418	97.2	95.6–98.8	359	83.5	79.9–87.1	240	55.8	51.0–60.6

*CI* confidence interval, *N* number of patients

**Table 3:** The geometric mean titer (GMT) level for all diseases across the different age groups enrolled in this study

Age (years)	Total	GMT level (IU/ml)											
		Diphtheria				Tetanus				Pertussis			
		*N*	GMT	95% CI	*p* value^†^	*N*	GMT	95% CI	*p* value^†^	*N*	GMT	95% CI	*p* value^†^
61–65	104	101	0.11	0.09–0.15	0.59	90	0.45	0.28–0.72	0.15	53	12.36	10.24–14.93	0.07
66–70	99	96	0.13	0.09–0.17		83	0.48	0.31–0.76		57	12.40	10.18–15.11	
71–75	92	88	0.11	0.09–0.14		72	0.24	0.14–0.39		48	12.46	10.27–15.13	
76–80	70	69	0.15	0.11–0.20		63	0.34	0.19–0.61		44	17.81	13.68–23.17	
81+	65	64	0.11	0.08–0.15		51	0.24	0.12–0.44		38	15.08	11.56–19.68	
Total	430	418	0.12	0.11–0.14		359	0.35	0.28–0.44		240	13.67	12.42–15.06	

*GMT* geometric mean titer

^†^Using the one-way-ANOVA

The GMT was calculated from the anti-diphtheria toxoid concentration > 0.01 IU/ml, which indicated seropositivity, as shown in Table 2. The GMT shown in Table 3 shows similarity across all age groups (0.11–0.15 IU/ml). The highest GMT was found among subjects aged 76–80 years (GMT 0.15 IU/ml). This was followed by those aged 66–70 years (0.13 IU/ml). The lowest GMT (0.11 IU/ml) was identified in three age groups (61–65, 71–75, and 81+ years).

When samples were classified according to antibody level, the majority of the population had antibody levels > 0.01 IU/ml, 45.8% had low immunity, and only 7.2% had long-lasting immune protection (Fig. 5).

### Anti-tetanus toxoid antibody

**Fig. 3:**
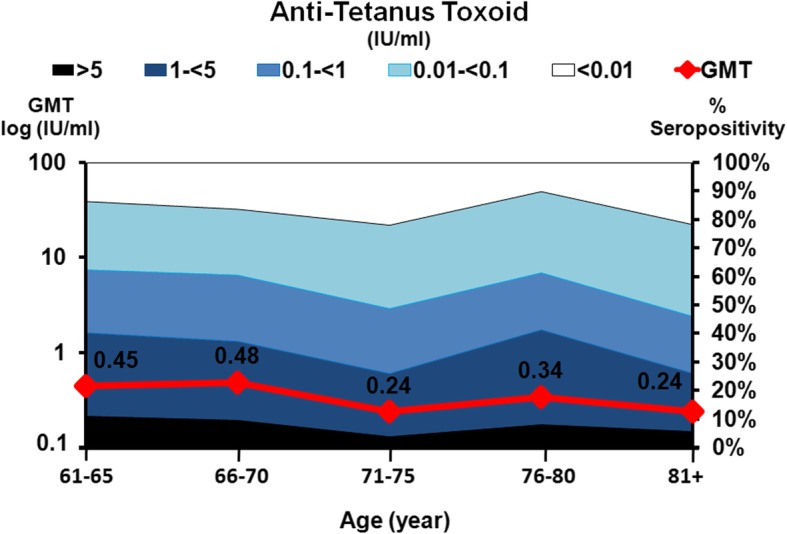
The prevalence of anti-tetanus toxoid antibody and geometric mean titers (GMTs) in the study population. The *x*-axis represents the five age groups and the sample size in each age group. The scale on the right represents the proportion (%) of the population with positive anti-tetanus toxoid antibody. The scale on the left represents the GMTs in each age group with the means indicated as a red line. Antibody measurements were negative (white), 0.1to < 0.5 IU/ml (light blue), 0.5 to < 1.1 IU/ml (blue), 1.1 to < 5 IU/ml (dark blue), and > 5 IU/ml (black)

Anti-tetanus toxoid antibody levels in all age groups are shown in Fig. 3 and Table 2; GMT for this antibody is shown in Table 3. The minimum protective level along with seropositivity is considered to be 0.01 IU/ml, and durable antibody protection levels (DAPL) are ≥ 1 IU/ml [19].

The GMT was calculated from the anti-tetanus toxoid concentration > 0.01 IU/ml, which indicated seropositivity, as shown in Table 2. The GMT of anti-tetanus toxoid antibody in all age groups was not significantly different and varied between 0.24 and 0.48 IU/ml (Table 3). The highest GMT was found in subjects aged 66–70 years (GMT 0.48 IU/ml) while the lowest GMT was found in those aged 71–75 years and 81+ years (GMT 0.24 IU/ml). Approximately 83.5% of the elderly population studies had sufficient immunity to protect themselves from tetanus, as indicated in Table 2.

When samples were classified according to antibody level, we found that the majority of the population had antibody levels > 0.01 IU/ml and approximately 34% of subjects had durable antibody protection levels (DAPL) ≥ 1 IU/ml (Fig. 5). Only 16.5% of the population were seronegative.

### Anti-*Bordetella pertussis* toxin antibody

**Fig. 4:**
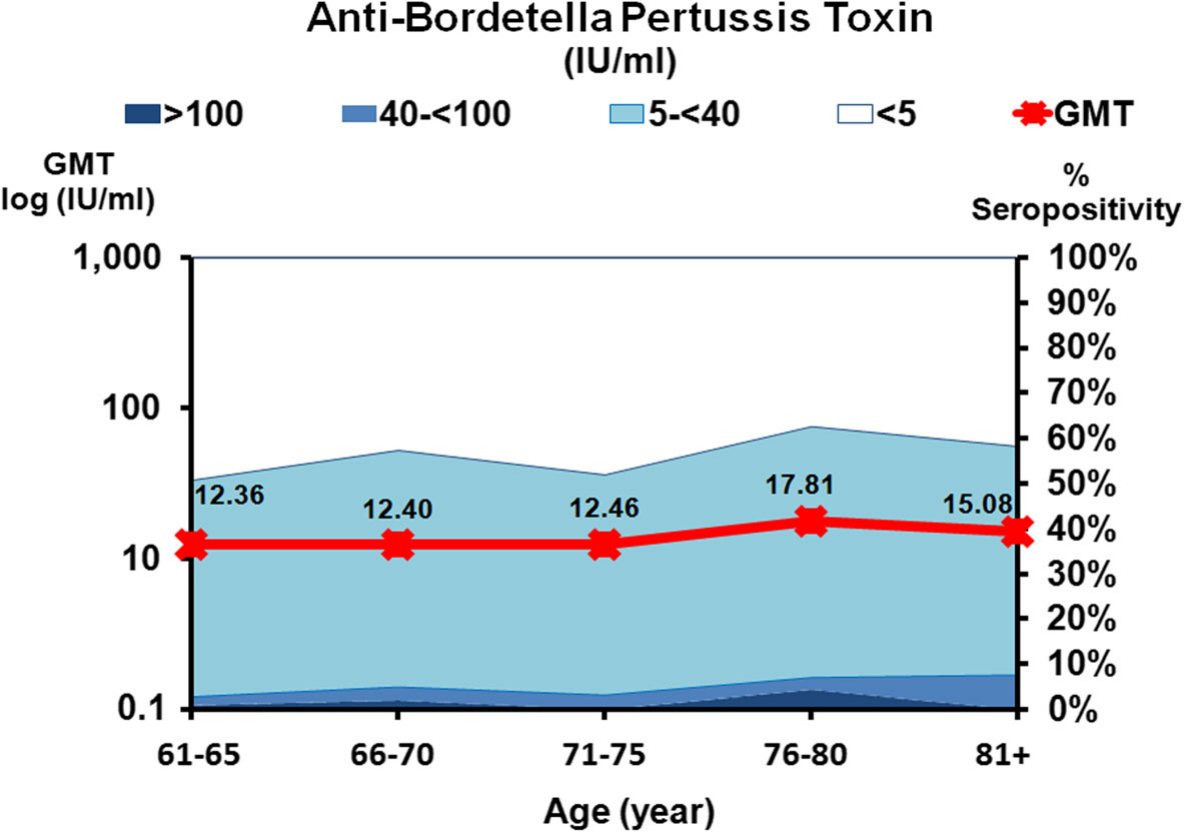
The prevalence of anti-*Bordetella pertussis* toxin antibody and geometric mean titers (GMTs) in the study population. The *x*-axis represents the five age groups and the sample size in each age group. The scale on the right represents the proportion (%) of the population with positive anti-*Bordetella pertussis* toxin antibody. The scale on the left represents the GMTs in each age group, with the means indicated as a red line. Antibody measurements were negative (white), 5 to < 40 IU/ml (light blue), 40 to < 100 IU/ml (blue), and >100 IU/ml (dark blue)

Anti-pertussis toxin antibody levels in all age groups are shown in Fig. 4 and Table 2; GMT is shown in Table 3. For anti-pertussis toxin, values < 5 IU/ml were interpreted as seronegative, 5–40 IU/ml as no evidence of recent acute infection, 40–100 IU/ml as probable past exposure to pertussis, and > 100 IU/ml as acute pertussis infection or recent vaccination [13, 21].

The GMT was calculated from an anti-*Bordetella pertussis* toxin concentration > 5 IU/ml, which indicated seropositivity, as shown in Table 2. In a manner similar to anti-tetanus toxoid antibody, the GMT of anti-*Bordetella pertussis* toxin antibody across all age groups was not significantly different and varied between 12.36 and 17.81 IU/ml (Fig. 4 and Table 3). The highest GMT was found in subjects aged 76–80 years (GMT 17.8 IU/ml) while the lowest GMT was found in those aged 61–65 years (GMT 12.36 IU/ml).

**Fig. 5:**
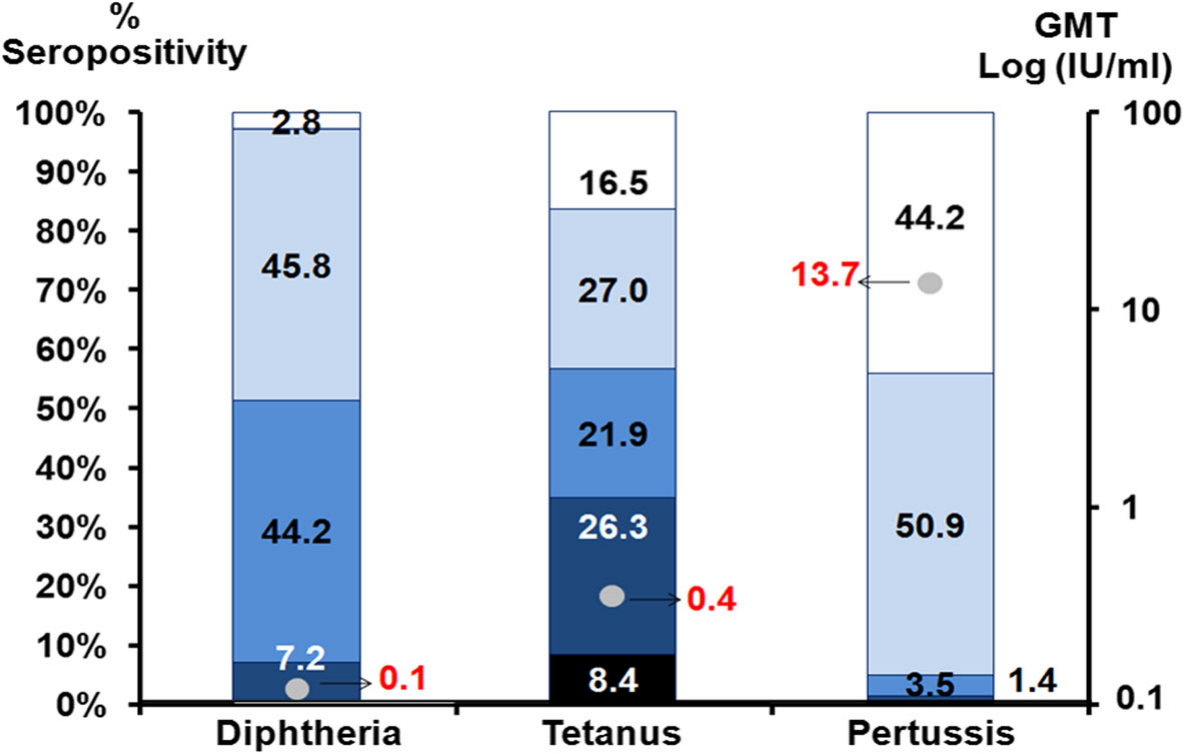
The proportion (%) of seropositive anti-toxin levels for diphtheria, tetanus, and pertussis across the study population enrolled in this study. The GMT is shown as red dots. Diphtheria toxoid antibody measurements were negative (white), 0.01 to < 0.1 IU/ml (light blue), 0.1 to < 1 IU/ml (blue), and > 1 IU/ml (dark blue). Tetanus toxoid antibody measurements were negative (white), 0.01 to < 0.1 IU/ml (light blue), 0.1 to < 1 IU/ml (blue), and 1 to < 5 IU/ml (dark blue), and > 5 IU/ml (black). Pertussis toxin antibody measurements were negative (white), 5 to < 40 IU/ml (light blue), 40 to < 100 IU/ml (blue), and > 100 IU/ml (dark blue)

When samples were classified according to antibody level, nearly 45% of the population had an antibody level lower than the seropositivity level (Fig. 5). Approximately 3.5% and 1.4% of the population showed probable past exposure to pertussis, acute infection, or recent vaccination.

## Discussion

The number of elderly people (defined as ≥ 60 years) in Thailand has grown rapidly due to the declining birth rate and a longer lifespan related to more advanced technology in the medical and healthcare sector. Moreover, the number of elderly people in Thailand will continue to rise and is projected to increase to more than 20 million over the next two decades [1, 2].

The elderly in Thailand can be divided into three groups: well elderly or socially bound elderly; homebound elderly; and bed-bound elderly [22]. The socially bound elderly cohort can perform all activities of daily living by themselves and can socialize with groups of the same age such as senior citizen clubs, volunteer groups, and recreation groups. The homebound elderly cohort has some limitations in performing some activities, so they do not like to go out of the house or have some duties to carry out at home, such as taking care of their grandchildren, cooking, cleaning, and looking after the house. In contrast, the bed-bound elderly cannot do anything by themselves and can only stay in bed or sit in a wheelchair. Most of the participants enrolled in the present study are classified as socially bound elderly (male) and homebound elderly (female). If some infectious diseases outbreak, the socially bound elderly can transmit the diseases to their family members. We should therefore be concerned about the immunity of the elderly population to many diseases in order to help them to resist the diseases.

In terms of diphtheria immunity, as determined in the present study, more than 95% of our population had an antibody level higher than the seronegativity level (> 0.01 IU/ml). Based on the hypothesis of “herd immunity,” diphtheria immunity among up to 85% of the population is required to inhibit an outbreak of diphtheria [23]. Booster administration should therefore be carried out for the elderly in future to maintain antibody levels because several studies have shown that protection levels decrease with increasing age [20, 24–27].

Tetanus is a rare disease but has a high mortality rate. The elderly are at significant risk of tetanus because they have never been immunized to this disease or because their immunity has waned. With regard to tetanus immunity, our present study found that 16.5% of our study population had an antibody level lower than seronegativity level (< 0.01 IU/ml). This finding may also be due to lack of immunization in this group since they had been born before the introduction of tetanus immunization into Thailand’s national immunization program. This finding concurs with previous studies in that the protective tetanus antibody levels decrease with increasing age [3, 19, 28]. According to this study, the population above 60 years of age should be targeted for reimmunization, especially if they sustain any tetanus-prone injury; this is because herd immunity does not occur in tetanus [19].

In terms of pertussis immunity, our study found that the seronegative rate was 44.2% among the elderly subjects who were born before the EPI, or those who had received fewer than three doses of pertussis vaccine. These observations suggest that antibody titers from vaccine-induced immunity do not last long, and in the pre-vaccination era when the elderly population was exposed to *B. pertussis*, antibody levels did not remain positive and high throughout the lifetime. The seronegative rate in this study is very similar to a previous study involving adolescents [13]. The highest GMT for pertussis was found in the group aged 76–80 years, for which protective immunity may have arisen from natural infections from the community. Since undiagnosed infected adults may spread a disease organism to non-immune individuals, infections in some high-risk groups, including young children, can be more severe and potentially fatal. From this study, we found that the protective immunity to pertussis was quite low among the elderly population, who represent the majority of Thai society in the future. The national vaccination program in Thailand must therefore be emphasized with regard to its role for routine pertussis vaccination in elderly adults as well as in infants and children; this practice will create herd immunity in the population [29].

There are some limitations to this study. Firstly, participants in this study were from one district in Thailand which may not represent the whole country. Secondly, there was female predominance in the study participants (male 28.6%, female 71.4%) possibly because males were not present during the daytime home visits for blood sampling, but the number of both groups was not significantly different. However, the female predominance in the elderly age group correlates with the national data for higher life expectancy in females compared to males. In addition, the antibody level (GMT) of the three diseases of male and female was not significantly different in diphtheria and pertussis. For tetanus, the antibody level in female was more statistically significant than that in male (0.67 vs 0.30, *p* value < 0.01 data not shown).

As mentioned above, the elderly group has an important role in the aging society of Thailand. Most of these subjects still have activities with their groups or take care of their grandchildren. Consequently, they can transmit and spread some diseases to the community, and their home, especially if they have low immunity to some diseases such as diphtheria, pertussis, and tetanus (DPT), as shown in this study. The Thai Government should therefore be concerned about these problems and provide revaccination to the elderly in order to promote their immunity to these diseases, as recommended in many other countries [30]. For example, Tdap revaccination not only increases herd immunity to diphtheria in the population, but also protects newborn infants from pertussis by the cocoon strategy, while also protecting the elderly from tetanus-prone injury.

## Data Availability

The datasets used and/or analyzed during the current study are available for scrutiny. Please contact the corresponding author to obtain a data access request.

## Abbreviations

DAPL: Durable antibody protection levels
ELISA: Enzyme-linked immunosorbent assay
EPI: Expanded Program on Immunization
GMT: Geometric mean titer
IRB: Institutional Review Board
Tdap: A tetanus toxoid, reduced diphtheria toxoid, and acellular pertussis vaccine
